# Distinct concentration-dependent oxidative stress profiles by cadmium in a rat kidney proximal tubule cell line

**DOI:** 10.1007/s00204-023-03677-z

**Published:** 2024-01-30

**Authors:** Wing-Kee Lee, Stephanie Probst, Bettina Scharner, Timo Deba, Faouzi Dahdouh, Frank Thévenod

**Affiliations:** 1 Institute of Physiology, Pathophysiology and Toxicology, ZBAF, Witten/Herdecke University, Stockumer Str. 12, 58453 Witten, Germany; 2 Physiology and Pathophysiology of Cells and Membranes, Medical School OWL, Bielefeld University, Morgenbreede 1, 33615 Bielefeld, Germany; 3 Present Address: Department of General Paediatrics, Klinik für Kinder- und Jugendmedizin, University Hospital Münster, Albert-Schweitzer-Campus 1, 48149 Münster, Germany; 4 Present Address: Department of Natural Sciences, Higher School of Professors for Technological Education, Skikda, Algeria

**Keywords:** Hydrogen peroxide, Superoxide, Catalase, Redox, Reactive oxygen species

## Abstract

Levels and chemical species of reactive oxygen/nitrogen species (ROS/RNS) determine oxidative eustress and distress. Abundance of uptake pathways and high oxygen consumption for ATP-dependent transport makes the renal proximal tubule particularly susceptible to cadmium (Cd^2+^)-induced oxidative stress by targeting ROS/RNS generation or antioxidant defence mechanisms, such as superoxide dismutase (SOD) or H_2_O_2_-metabolizing catalase (CAT). Though ROS/RNS are well-evidenced, the role of distinct ROS profiles in Cd^2+^ concentration-dependent toxicity is not clear. In renal cells, Cd^2+^ (10–50 µM) oxidized dihydrorhodamine 123, reaching a maximum at 2–3 h. Increases (up to fourfold) in lipid peroxidation by TBARS assay and H_2_O_2_ by Amplex Red were evident within 30 min. ROS and loss in cell viability by MTT assay with 50 µM Cd^2+^ could not be fully reversed by SOD mimetics Tempol and MnTBAP nor by SOD1 overexpression, whereas CAT expression and α-tocopherol were effective. SOD and CAT activities were attenuated below controls only with >6 h 50 µM Cd^2+^, yet augmented by up to 1.5- and 1.2-fold, respectively, by 10 µM Cd^2+^. Moreover, 10 µM, but not 25–50 µM Cd^2+^, caused 1.7-fold increase in superoxide anion (O_2_^•−^), detected by dihydroethidium, paralled by loss in cell viability, that was abolished by Tempol, MnTBAP, α-tocopherol and SOD1 or CAT overexpression. H_2_O_2_-generating NADPH oxidase 4 (NOX4) was attenuated by ~50% with 10 µM Cd^2+^ at 3 h compared to upregulation by 50 µM Cd^2+^ (~1.4-fold, 30 min), which was sustained for 24 h. In summary, O_2_^•−^ predominates with low–moderate Cd^2+^, driving an adaptive response, whereas oxidative stress by elevated H_2_O_2_ at high Cd^2+^ triggers cell death signaling pathways.

Highlights

- Different levels of reactive oxygen species are generated, depending on cadmium concentration.
- Superoxide anion predominates and H_2_O_2_ is suppressed with low cadmium representing oxidative eustress.
- High cadmium fosters H_2_O_2_ by inhibiting catalase and increasing NOX4 leading to oxidative distress.
- Superoxide dismutase mimetics and overexpression were less effective with high versus low cadmium.
- Oxidative stress profile could dictate downstream signalling pathways.

**Supplementary Information:**

The online version contains supplementary material available at 10.1007/s00204-023-03677-z.

## Introduction

Most living organisms cannot exist without oxygen. During oxygen metabolism, reactive oxygen species (ROS) and reactive nitrogen species (RNS) are created as by-products. In mammalian cells, the majority of cellular ROS is continuously produced from oxygen-consuming mitochondria through the electron transport chain (ETC) and oxidative phosphorylation during ATP production. Other key endogenous sources of ROS are NADPH oxidases (NOXs), peroxisomes, lysosomes and the endoplasmic reticulum. Thus, the presence of ROS is physiological (Sies and Jones 2020). However, ROS/RNS are highly reactive in nature and must be rapidly removed by antioxidative defence mechanisms to prevent oxidative stress and cellular damage. Indeed, excess ROS/RNS production also forms part of the cellular stress and adaptive response to exogenous stimuli, such as drugs and toxic metals, and can cause cytotoxicity and potentially deleterious effects at high levels through damage to DNA, lipids and proteins.

Reactions with molecular oxygen generate superoxide anions (O_2_^•−^) that can be metabolized to other ROS/RNS, such as hydrogen peroxide (H_2_O_2_) and hydroxyl radicals (•OH), as well as reacting with nitric oxide (NO) to generate peroxynitrite (ONOO^−^). Levels of ROS/RNS are kept to a minimum by detoxifying mechanisms, including superoxide dismutases (SODs), catalase (CAT), thioredoxins, peroxidases as well as enzymes responsible for the synthesis and reduction of the peptide glutathione (GSH) (Halliwell and Gutteridge 2007; Haugaard 1968). Superoxide anions are metabolized by three isoforms of SOD in humans: SOD1 is localized in the cytosol and is dependent on copper and zinc; SOD2 is found in mitochondria and requires manganese as a co-factor; SOD3 is located on the extracellular cell surface and also contains copper and zinc in its reactive centre. SODs catalyse the dismutation of superoxide anions to H_2_O_2_, which is consequently detoxified to oxygen and water by the enzymatic actions of CAT, primarily localized in peroxisomes, yet also found in the cytosol, or to water by glutathione peroxidases (GPx), which are most abundant in the cytosol.

Despite the potentially damaging effect of excessive ROS/RNS, it is becoming increasingly apparent that they also function as signaling molecules, depending on the amount and type of ROS/RNS generated. High global levels of ROS/RNS initiate cell death, whereas low global levels and/or localized changes in ROS/RNS can be integrated in redox signal transduction and/or induce cellular adaptation responses. To this end, numerous reports have indicated that physiological ROS/RNS levels are required for cell proliferation and cell cycle progression as well as governing cellular life and death decisions (Bae et al. 2011; Sies et al. 2022; Sies and Jones 2020).

The toxic metal cadmium (Cd^2+^) is released from the Earth’s crust into the environment primarily by human industrial activities, resulting in contamination of wastewaters, uptake into plants and food sources and posing a human health hazard (IARC 2012). Upon entering the human body, Cd^2+^ is detoxified mainly by complexation with metallothionein (Sabolic et al. 2010), but also with other proteins, for example, β2-microglobulin, or with peptides, such as GSH (Perrin and Watt 1971). Pertinent to their small sizes, these Cd^2+^-complexes are easily filtered by the kidney and are removed from the primary filtrate by receptor-mediated endocytosis or are cleaved at the luminal brush border membrane and ionic Cd^2+^ is taken up by divalent cation transporters, such as those for zinc or iron, in the proximal tubule (PT) (Adamis et al. 2009; Shafer 2000; Thévenod and Lee 2013b). Renal damage by Cd^2+^ occurs primarily in the PT due to the plethora of cellular entry modalities present in concert with high O_2_ consumption/mitochondrial density due to transport activity (Thévenod et al. 2019; Wirthensohn and Guder 1986). Should these Cd^2+^-complexes escape the PT, they are taken up by the distal nephron, where protein endocytosis is mediated in part by the lipocalin-2 receptor (SLC22A17) (Langelueddecke et al. 2012). Once inside the cell, Cd^2+^ is complexed and accumulates over 20–30 years. Once detoxification capacity is exceeded, renal damage and/or cancer transformation may occur (Johri et al. 2010; Schwerdtle et al. 2010).

The increased formation of ROS/RNS by Cd^2+^ is essential to its multifaceted cellular effects and has been summarized in a number of excellent reviews (Cuypers et al. 2010; Kitamura and Hiramatsu 2010; Liu et al. 2009; Thévenod 2009). Multiple mechanisms can be affected by Cd^2+^ to increase ROS/RNS levels: displacement of Fenton metals (O'Brien and Salacinski 1998), inhibition of the mitochondrial ETC (Wang et al. 2004), decrease of antioxidant enzyme activities (Waisberg et al. 2003), reduction of GSH levels (L'Hoste et al. 2009) and activation of NOXs (Souza et al. 2009). In the current study, the importance of Cd^2+^ concentration in determining the type and levels of generated ROS is evidenced in the WKPT-0293 Cl.2 cell line, derived from the S1-segment of the rat renal proximal tubule (PT) (Woost et al. 1996), which is the primary site for Cd^2+^ uptake and injury in the kidney (Thévenod and Lee 2013a; b). Low–moderate Cd^2+^ concentrations, reflecting subacute exposure, accumulation, and chronic disease progression associated with Cd^2+^, stimulate low levels of ROS dominated by superoxide anion, whereas high Cd^2+^ concentrations, similar to acute exposure of large amounts of Cd^2+^, block CAT and produce large ROS levels, mostly H_2_O_2_, creating oxidative distress. The generation of distinct ROS species by Cd^2+^ affects downstream signaling pathways and ultimately impacts the outcome in cellular behaviour and cell fate.

## Materials and methods

### Materials

Dihydrorhodamine 123 (DHR123), manganese [III] tetrakis (4-benzoic acid) porphyrin (MnTBAP), diphenyleneiodonium chloride (DPI) and catalase fluorometric detection kit were from Enzo Life Sciences (Farmingdale, NY). CdCl_2_ was from Merck (Nottingham, UK). Dihydroethidium was obtained from Cayman Chemical Company (Ann Arbor, MI). MTT, Tempol, sulfosalicylic acid (SSA), apocynin and α-tocopherol (vitamin E) were from Sigma-Aldrich (Deisenhofen, Germany). FAST SYBR Green 2X Mastermix was purchased from Applied Biosystems (Carlsbad, CA) or from KAPA Biosystems, Inc. (Roche, Basel, Switzerland). PSC833 was a gift from Sanofi-Aventis, Basel, Switzerland. Primers were obtained from Eurofins MWG Operon (Ebersberg, Germany). All other chemicals were from commercial sources and of the highest purity available.

## Methods

### Cell culture and treatment

The SV40 antigen immortalized cell line WKPT-0293 Cl.2 derived from the S1 segment of rat kidney PT was cultured as previously described (Lee et al. 2005a). Cells were grown for two days prior to treatment and incubations with inhibitors or CdCl_2_ (Cd^2+^) were performed in serum free medium (SFM), unless otherwise stated.

### Plasmids and transfection

Plasmids coding for human superoxide dismutase 1 (pSG5-SOD1) and human catalase (pcDNA3.1-CAT) were kind gifts from Dr. J. Dulak (Jagiellonian University, Kraków, Poland) (Grzenkowicz-Wydra et al. 2004) and Dr. N. Akiyama (RIKEN, Saitama, Japan) (Nishikawa et al. 2004), respectively. Plasmids were introduced into WKPT-0293 Cl.2 cells using Lipofectamine 2000 (Invitrogen) following manufacturer’s instructions.

### ROS/RNS detection using DHR123

DHR123 is oxidized to fluorescent rhodamine 123^+^ (Rh123^+^), which accumulates in the mitochondrial matrix. Following treatment, cells were incubated with 2.5 µM DHR123 + 1 µM PSC833 (an inhibitor of ABCB1 to improve dye loading) in Hank’s buffered salt solution (HBSS) for 30 min at 37 °C. Cells were washed twice with HBSS and lysed in RIPA buffer. Intracellular Rh123^+^ fluorescence was determined on a Berthold Mithras LB940 at λ_ex_/λ_em_ 485/535 nm. For fluorescence imaging, cells in 35 mm dishes were imaged immediately after washing with HBSS on a Zeiss Axiovert 200M inverted microscope illuminated with a mercury HBO lamp or Sola SM II light engine (Lumencor) and equipped with Fluar 40x/N.A. 1.3 and 100x/N.A. 1.3 oil immersion objectives and Cool-SNAP ES CCD camera (Roper Scientific). Images were acquired at fixed exposure times at the focal plane of highest contrast and analyzed with FIJI (Schindelin et al. 2012).

### H_2_O_2_ detection using Amplex Red

H_2_O_2_ was detected using the Amplex Red assay (Molecular Probes) following manufacturer’s instructions. Cells were lysed by three cycles of freeze/thaw followed by centrifugation at 1000×*g* for 3 min at 4 °C to remove unbroken cells. Supernatants were used in the assay, which consisted of 0.2 U/ml horseradish peroxidase and 100 µM Amplex Red reagent. After 30 min, Amplex Red fluorescence was measured at λ_ex_/λ_em_ 535/590 nm. Protein concentrations were determined by the method of Bradford (Bradford 1976).

### TBARS assay

Thiobarbituric acid reactive substances (TBARS) are by-products of lipid peroxidation. Cells (8 × 10^5^ seeded in a 25 cm^2^ flask) were treated, harvested by scraping into PBS and pelleted at 3000×*g* for 3 min at 4 °C. TBARS assay was performed using the Lipid Peroxidation Assay Kit (MAK085, Sigma-Aldrich), according to manufacturer’s instructions. Cell pellets were resuspended in lysis buffer containing butylated hydroxytoluene on ice and were centrifuged at 15,000×*g* for 10 min. Supernatants were mixed with thiobarbituric acid, incubated at 95 °C for 60 min and the reaction was stopped by cooling on ice. Following filtration of samples to remove precipitates, fluorescence was measured at λ_ex_/λ_em_ 530/590 nm in a Berthold Mithras LB940 microplate reader. The MDA concentration in unknown samples was calculated using a standard curve and reported as % non-treated controls.

### MTT assay

Conversion of MTT to its formazan product was used as a measure of cell viability, and therefore cytotoxicity. The assay was performed as previously described (Lee et al. 2005a).

### Superoxide anion detection using dihydroethidium

WKPT-0293 Cl.2 cells (2.5 × 10^4^ per well) were plated and grown in 24-well plates prior to Cd^2+^ treatment. Cells were washed twice with HBSS, 10 µM dihydroethidium (DHE) was added and incubated for 30 min at 37 °C. For plate reader studies, cells were washed thrice with HBSS and intracellular fluorescence was measured at λ_ex_/λ_em_ 535/590 nm and 380/590 nm in a Berthold Mithras LB940. For microscopy, cells were fixed in 2% paraformaldehyde (PFA) for 10 min, nuclei were counterstained with 0.8 µg/ml Hoechst-33342 and visualized at λ_ex_/λ_em_ 350/460 nm and 518/605 nm for Hoechst and DHE, respectively, on the imaging system described above.

### Superoxide dismutase activity

After treatment, cells (3 × 10^5^ plated per 6 well) were harvested by scraping into PBS and centrifuged at 3000×*g* for 3 min at 4 °C. Pellets were resuspended in lysis buffer (20 mM Hepes, 1 mM EGTA, 210 mM mannitol, 70 mM sucrose, pH 7.2) and cells were homogenized by sonication. Unbroken cells were removed by centrifugation at 1500×*g* for 5 min at 4 °C. SOD activity was determined using the Superoxide Dismutase Assay Kit (#706002, Cayman Chemical Company) following manufacturer’s instructions. Samples were diluted 1:10 in lysis buffer, combined with a radical detector and xanthine oxidase, and incubated at shaking at room temperature for 30 min. Absorption was measured at 450 nm in a Berthold Mithras LB940. Protein concentration was determined by Bradford assay. SOD activity was calculated using a standard curve, corrected for protein and normalized to non-treated controls.

### Catalase activity

Analysis of catalase enzymatic activity was performed according to manufacturer’s instructions (Catalase Fluorometric Kit ADI-907-027, Enzo Life Sciences). Cells were scraped into PBS and pelleted at 5000×*g* for 1.5 min at 4 °C. Pellets were resuspended in 1X reaction buffer and lysed by three cycles of freeze/thaw. Unbroken cells were removed by centrifugation at 1000×*g* for 3 min at 4 °C. Protein concentration was determined by Bradford assay. Catalase activity from 0.5 µg protein was assayed by incubation with 20 µM H_2_O_2_ for 60 min followed by detection reagent for 10 min and fluorescence was determined at λ_ex_/λ_em_ 535/590 nm in a Mithras LB940 plate reader (Berthold Technologies). Catalase activity from unknown samples was interpolated from a standard curve (0–62.31 U/ml), where one unit decomposes one µmole of H_2_O_2_ per min at pH 7.0 at 25 °C.

### Glutathione measurements

Reduced (GSH) and oxidized (GSSG) glutathione were measured according to manufacturer’s instructions (DetectX® Glutathione Colorimetric Detection Kit K006-H1, Arbor Assays). Cells were harvested by trypsinization and pelleting at 5000×*g* for 1.5 min at 4 °C. Pellets were washed once with PBS, resuspended in cold 5% SSA and lysed by three cycles of freeze/thaw. Samples were deproteinized by incubating for 10 min on ice and centrifuging at 16,100×*g* for 10 min at 4 °C. Supernatants were diluted 1:5. Standards and samples were incubated with Detection Reagent and Reaction Mixture for 20 min at room temperature and absorbance was determined at 405 nm in a Berthold Mithras LB940. Glutathione content from unknown samples was interpolated from a standard curve in the range of 0–25 µM. Protein content was measured from SSA precipitated protein by the method of Bradford.

### Quantitative real-time PCR

RNA was isolated from WKPT-0293 Cl.2 cells using High Pure RNA Isolation Kit (Roche) and cDNA was immediately synthesized from 2 µg RNA with oligo dT_18_ primers using First Strand cDNA Synthesis kit (Fermentas). Prior to use in PCR reactions, the cDNA mixture was diluted 1:10 with 1/10 TE buffer (1 mM Tris-HCl, 0.1 mM EDTA, pH 8.0) and used at 1/5 of total reaction volume. Real-time PCR was performed with FAST SYBR Green 2X Mastermix in an 7300HT Fast (Applied Biosystems) or with KAPA SYBR FAST qPCR Master Mix in a StepOnePlus (Applied Biosystems) PCR system using universal fast cycling conditions (activation at 95 °C for 20 s or 5 min followed by 40 cycles of 95 °C for 3 s and 60 °C for 30 s). Final melting curve analysis of PCR products was also performed. Primers were designed using NCBI Primer-BLAST program unless other stated (Supplementary Table s1). Primer efficiency tests and calculations using the formula: Efficiency = [10^(−1/slope)^ − 1] for each primer pair resulted in a score of 85–100%.

Ct values were derived from the exponential phase of the amplification plots (0.1 ΔRn). Minimum Ct from each experiment was subtracted from the Ct values, then converted using the formula 2^−ΔCt^ and expressed as relative expression to non-treated controls. Gene expression data were then normalized to multiple reference genes (Remans et al. 2008, Nair et al. 2015b). Suitability of reference genes was analyzed using the geNorm logarithm (Ghent University Hospital, Centre for Medical Genetics) (Vandesompele et al. 2002) for expression stability under the tested experimental conditions and a normalization factor was derived from the geometric mean of suitable reference genes.

### Reverse transcriptase semi-quantitative PCR

RNA and cDNA were isolated and generated as described above. PCR reactions were performed using Maxima Hot Start Green PCR Mastermix (Fermentas) with 50 ng cDNA and 500 nM primer. The reaction was started by activation at 95 °C for 5 min followed by 30 cycles of 94 °C for 20 s, 58 °C for 20 s and 72 °C for 30 s and a final elongation step at 72 °C for 10 min. PCR products were visualised on a 1.2% agarose gel loaded with GelRed nucleic acid stain (BioTrend) and imaged by UV light. Primer sequences are listed in Supplementary Table s2.

### Immunofluorescence staining

For NOX4 immunofluoresence staining, cells (2 × 10^4^ cells/24 well) were plated on glass coverslips, treated and fixed with 4% PFA in PBS for 10 min followed by permeabilization with 1% SDS for 5 min, and blocking with 1% bovine serum albumin in PBS (BSA–PBS) for 60 min. All steps were performed at room temperature. Nox4 antibody (1:100 in BSA–PBS, NB110-58851, Novus Biologicals) was incubated overnight at 4 °C and detected using Alexa Fluor 488 conjugated goat anti-rabbit IgG antibody (#A-11008, Thermo Fisher Scientific) diluted 1:500 and incubated for 1 h. Nuclei were counterstained with Hoechst-33342 for 5 min. Coverslips were embedded with fluorescent mounting medium (Dako). Images were acquired and analyzed as described above.

### Immunoblotting

Sample processing, SDS–PAGE and immunoblotting conditions have been reported elsewhere (Lee et al. 2005a). Equal amounts of protein (5 µg) were loaded and blotted with α-catalase antibody diluted 1:30,000, followed by species-specific horseradish peroxidase-coupled secondary antibody and developed with Immobilon enhanced chemiluminescence (Millipore). Signals were visualized on blue MXB X-ray films (Carestream) and densitometry analysis was performed using FIJI.

### Statistical analyses

Unless otherwise indicated, experiments were repeated at least three times and means ± SD are given. Standard curves from assay kits were fitted by nonlinear asymmetrical 5 point parametric curve fitting using GraphPad Prism and unknown samples were derived. Statistical significance was tested using unpaired Student’s *t* test for pairwise comparisons or one-way ANOVA with the appropriate post-hoc test for multiple comparisons (Dunn’s or Holm–Sidak). *p* values ≤ 0.05 were considered statistically significant, where * *p* < 0.05, ** *p* < 0.025, *** *p* < 0.01, **** *p* < 0.001.

## Results

### Cadmium increases ROS/RNS in a concentration-dependent manner

**Fig. 1:**
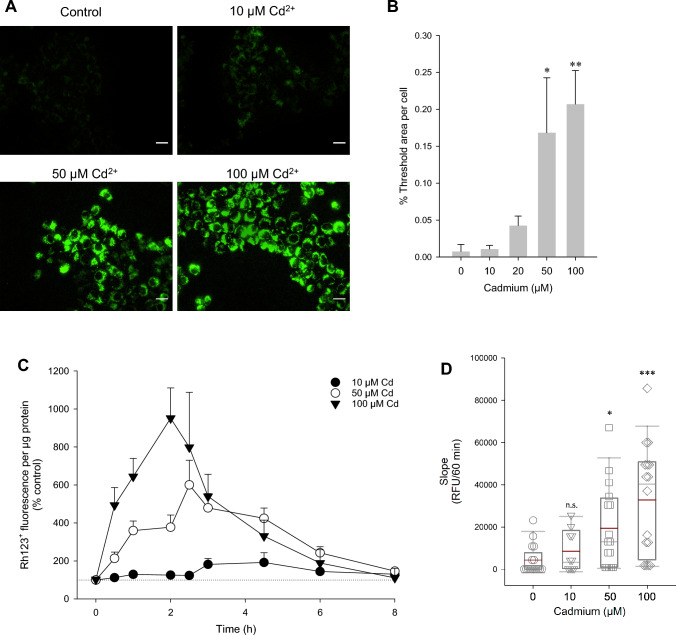

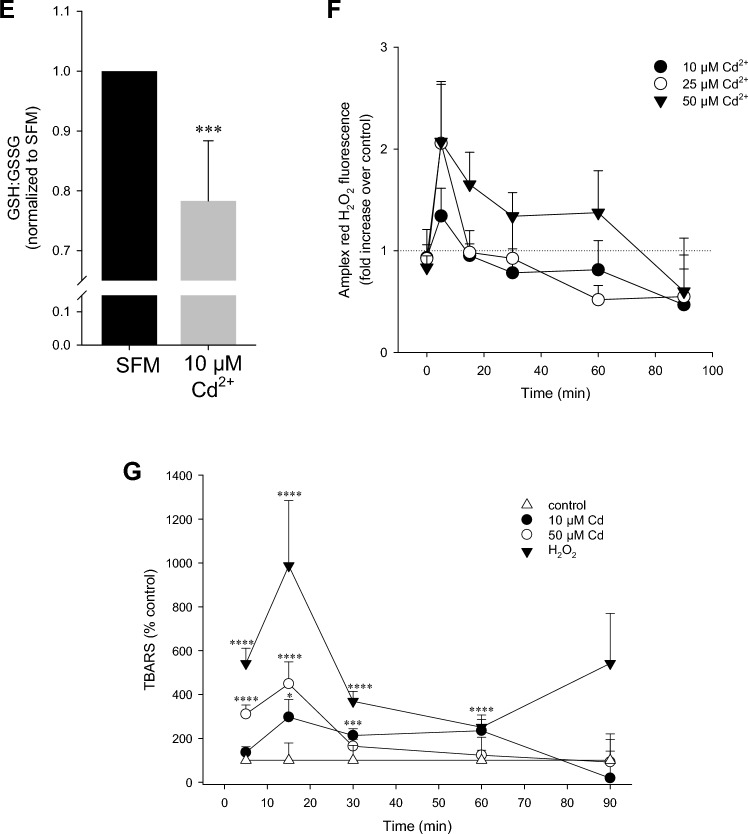
Oxidative stress and lipid peroxidation by Cd^2+^ in WKPT-0293 Cl.2 renal PT cells. Dihydrorhodamine 123 (DHR123) reacts with hydrogen peroxide (H_2_O_2_), hypochlorous acid (HOCl), and peroxynitrite anion (ONOO^−^) to form fluorescent rhodamine 123^+^ (Rh123^+^). **A** WKPT-0293 Cl.2 cells were exposed to varying Cd^2+^ concentrations for 1 h followed by ROS detection using DHR123. Images were acquired from non-fixed cells using a fluorescence imaging system. Scale bar = 20 µm. **B** Quantification of thresholded images from (**A**) from 4 to 5 independent experiments. **C** Kinetics of Cd^2+^-induced ROS generation using DHR123. After Cd^2+^ exposition at each timepoint, cells were incubated with DHR123, washed and lysed with RIPA buffer. Rh123^+^ fluorescence was determined in a microplate reader. **D** Slope analysis from the first 60 min of curves in (**C**) from *n* = 5–18. **E** Total cellular glutathione content. Reduced (GSH) and oxidized (GSSG) glutathione were determined after 3 h Cd^2+^ (*n* = 4). Unpaired two-tailed *t* test compares Cd^2+^ to SFM control. **F** Time course of H_2_O_2_. Measurements were performed after Cd^2+^ exposure at each timepoint using Amplex Red (*n* = 4–5). **G** Time course of lipid peroxidation. Measurements were performed after Cd^2+^ exposure at each timepoint using the TBARS assay (means ± SEM, *n* = 13–19). One-way ANOVA with Holm–Sidak posthoc test compares H_2_O_2_ or Cd^2+^-treated cells to non-treated controls [at each timepoint in (**G**)]

Though Cd^2+^ is not a Fenton metal, it is known to cause oxidative stress, based on damaging mitochondrial function or its ability to interfere with the function of ROS/RNS-generating and ROS/RNS-metabolizing enzymes (Cuypers et al. 2010). To determine the kinetics of ROS/RNS production by Cd^2+^, DHR123 was employed. DHR123 is oxidized to Rh123^+^ yet does not distinguish between H_2_O_2_, HOCl and ONOO^−^ nor does it detect superoxide anions (Spence and Johnson 2010). Fluorescence intensity of Rh123^+^ was elevated by 10–100 µM Cd^2+^ after 1 h using fluorescence imaging of non-fixed cells with concentrations ≥50 µM reaching statistical significance (Fig. 1A, B). Non-treated controls displayed little to no Rh123^+^ fluorescence. The intracellular punctate distribution indicates that Rh123^+^ has translocated to the mitochondrial matrix based on charge attraction (Emaus et al. 1986).

In time course studies, Rh123^+^ fluorescence was initially increased by Cd^2+^ peaking at 2–3 h, after which the intensity progressively dropped, returning to control levels after 8 h Cd^2+^ exposure (Fig. 1C). This biphasic response was independent of the Cd^2+^ concentration, though the peak occurred at later timepoints at lower Cd^2+^ concentrations (10 µM at 3–4.5 h versus 100 µM at 2 h). Slope analysis of the first hour of Cd^2+^ incubation shows significant increases for 50–100 µM Cd^2+^ but not for 10 µM Cd^2+^ (Fig. 1D) despite reduction in the GSH:GSSG ratio (Fig. 1E), as previously observed (Nair et al. 2015a). Since the cells are incubated with DHR123 at the end of Cd^2+^ incubation, and not during, the decrease in Rh123^+^ intensity at 4.5–8 h cannot be attributed to a quenching phenomenon. Without a doubt, ROS/RNS is induced by all Cd^2+^ concentrations though transient in nature, which was confirmed in H_2_O_2_ (Fig. 1F) and lipid peroxidation (Fig. 1G) studies.

### Generated species of ROS/RNS and cytotoxicity are dependent on Cd^2+^ concentration

**Fig. 2:**
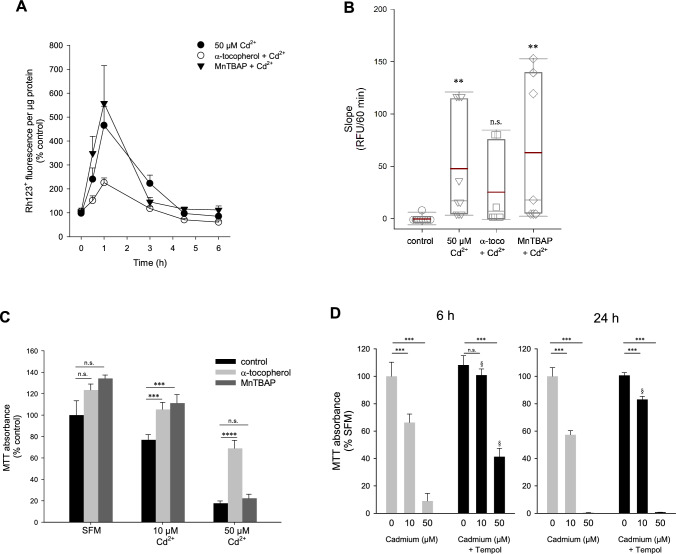
ROS by high Cd^2+^ cannot be rescued by superoxide dismutase mimetics. **A** WKPT-0293 Cl.2 cells were preincubated with 100 µM α-tocopherol or 100 µM MnTBAP for 1 h prior to Cd^2+^ addition. ROS were determined with DHR123 (*n* = 7–14). **B** Slope analysis of the first 60 min of the curves in (**A**). Box and whisker plots depict 5th and 95th percentile and the mean (solid line) (*n* = 7–14). **C** MTT cell viability assay of antioxidants ± 3 h Cd^2+^ treatment (*n* = 7–14). **D** WKPT-0293 Cl.2 cells were preincubated with 1 mM Tempol for 1 h prior to Cd^2+^ addition followed by MTT assay (*n* = 3–6). Pairwise comparisons using one-way ANOVA with Dunn’s test compares inhibitor ± Cd^2+^-treated cells to control (**B**), and with Holm–Sidak post hoc test compare antioxidant pre-treated cells to no antioxidant or Cd^2+^ only controls (**C**) and Cd^2+^ to controls as well as Tempol + Cd^2+^ to Cd^2+^ only samples (**D**; § = p < 0.01)

To gain better understanding of the differing response in ROS/RNS by low and high Cd^2+^ concentrations, antioxidants with distinct targets were utilized. The lipophilic general antioxidant α-tocopherol, a vitamin E, partitions into domains enriched in polyunsaturated phospholipids, preventing lipid oxidation (Atkinson et al. 2010). In contrast, MnTBAP, a synthetic metalloporphyrin, acts as a cell-permeable SOD mimetic but also scavenges peroxynitrite anions (Faulkner et al. 1994). Pre-incubation of PT cells with 100 µM α-tocopherol effectively prevented ROS/RNS accumulation by 50 µM Cd^2+^ (Fig. 2A). At 1 h, 50 µM Cd^2+^ increased Rh123^+^ intensity by 465.6 ± 80.1% (*n* = 8). This was reduced by more than half to 225.9 ± 64.9% (*n* = 7, *p* < 0.05) in the presence of 100 µM α-tocopherol, confirming our previous observations (Bork et al. 2010). Unexpectedly, 100 µM MnTBAP had no effect on Rh123^+^ intensity by 50 µM Cd^2+^ (557.9 ± 157.2%, *n* = 5, *p* = 0.57) (Fig. 2A). Similar observations were made with 10 µM Cd^2+^ (data not shown). Slope analysis of the first 60 min of the data in Fig. 2A reiterated Rh123^+^ increase by Cd^2+^ ± MnTBAP, whereas α-tocopherol is not significantly different from non-treated control (Fig. 2B). This was reflected in cytotoxicity induced by Cd^2+^ as determined by MTT assay. Increased cell death by 50 µM Cd^2+^ was significantly attenuated by α-tocopherol, similar to previous observations (Lee et al. 2012), but MnTBAP was ineffective (Fig. 2C). Interestingly, both antioxidant compounds could abolish cell death by 10 µM Cd^2+^ (Fig. 2C). In presence of the superoxide anion scavenger and SOD mimetic Tempol, loss in MTT absorbance by 10 µM Cd^2+^ was abolished at both 6 and 24 h (Fig. 2D). Conversely, decreased cell viability with 50 µM Cd^2+^ could not be completely reversed by Tempol although a partial effect was observed at 6 h (Fig. 2D). The different effects of the inhibitors could be explained by the antioxidative capacity of each compound, with α-tocopherol more effective than MnTBAP or Tempol, or it could indicate that different types of ROS/RNS are induced, depending on the concentration of Cd^2+^.

### Superoxide anions are generated upon exposure to low Cd^2+^

**Fig. 3:**
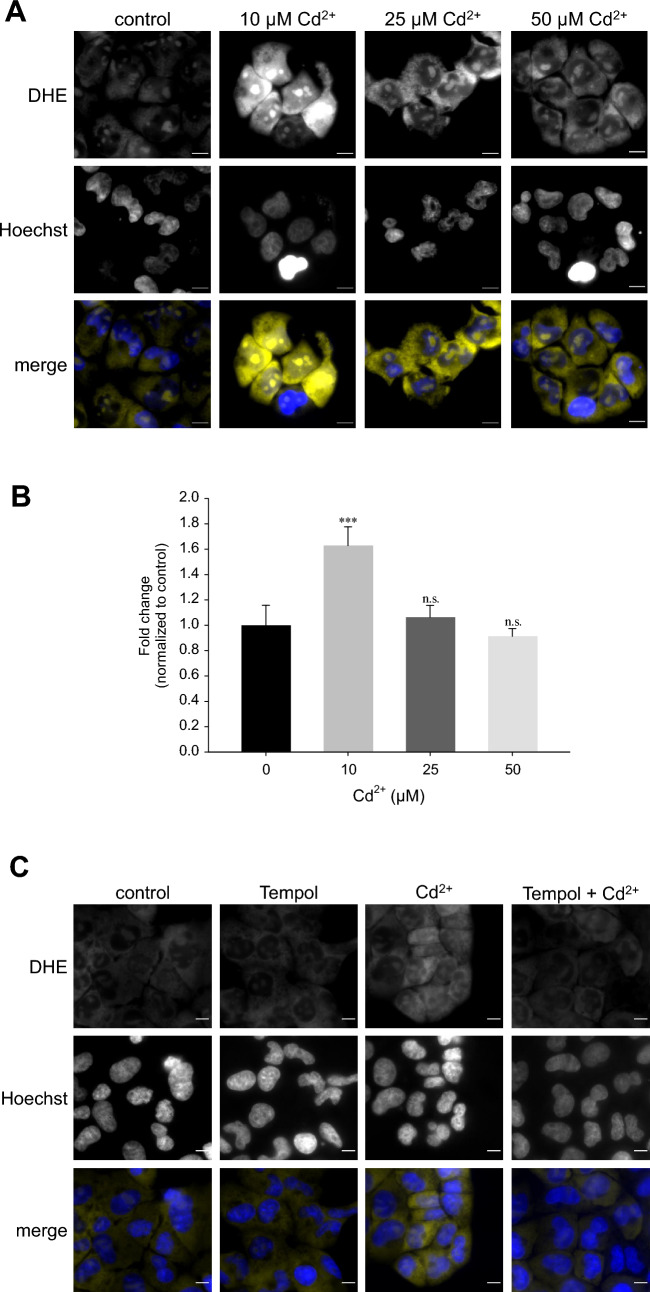

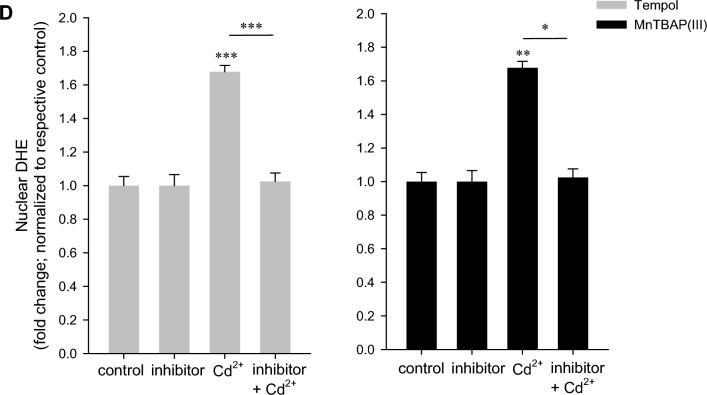
Superoxide anions predominate at low Cd^2+^. WKPT-0293 Cl.2 cells were exposed to Cd^2+^ for 1 h and superoxide anions were subsequently detected by dihydroethidium (DHE), microscope analysis (**A**) (scale bar = 20 µm) and quantification of thresholded images (**B**) (*n* = 5). **C** Pre-treatment with Tempol abolished superoxide anion generation by 10 µM Cd^2+^. Scale bar = 20 µm. Images are representative of 5 independent experiments. **D** Quantification of DHE oxidation in the nucleus with Tempol (*n* = 5) or MnTBAP (*n* = 9). Statistical analyses using one-way ANOVA with Holm–Sidak posthoc test compares Cd^2+^ treated to control cells (**B**) or antioxidant+Cd^2+^ to Cd^2+^ only cells (**D**)

Based on the above observations, we hypothesized the generation of predominantly superoxide anions by low-intermediate Cd^2+^ (<10 µM), whereas at higher Cd^2+^ (25–50 µM), H_2_O_2_ is prevalent. To discern different ROS species, DHE, which is oxidized to fluorescent (2-hydroxy)ethidium by superoxide anions was employed (Spence and Johnson 2010). Though ethidium (300/600 nm excitation/emission maxima) is often used to assess oxidation of DHE, it is not specific for superoxide anions and can be the oxidation product of other ROS/RNS, whereas 2-hydroxyethidium is considered as an oxidation product attributed to superoxide anions and can be measured by excitation at 520 nm (Zhao et al. 2003). As shown in Fig. 3A, DHE exhibits little fluorescence under control conditions. Application of Cd^2+^ for 1 h resulted in an approximately 1.8-fold increase in fluorescence, which was significant only for 10 µM Cd^2+^ and not higher concentrations (Fig. 3B). In more detailed studies, the oxidation products were measured in lysed cells using a microplate reader. The less-specific ethidium was measured at 380/590 nm, whereas 2-hydroxyethidium was determined at 535/590 nm. Following these detection criteria, only 10 µM Cd^2+^ significantly increased 2-hydroxyethidium. In contrast, ethidium was augmented at all Cd^2+^ concentrations tested (Supplementary Figure 1). To confirm increased fluorescence signals by microscopy are attributed to superoxide anions, cells were preincubated with SOD mimetics Tempol or MnTBAP prior to incubation with 10 µM Cd for 1 h. Nuclear 2-hydroxyethidium signals were determined by region analysis and were abolished by both compounds (Fig. 3C, D), confirming superoxide-specific signals and generation of superoxide anions is associated with lower Cd^2+^ concentrations. Taken together, this set of studies indicates superoxide anion generation upon low-intermediate Cd^2+^ exposure, whereas high Cd^2+^ levels could be damaging to superoxide-generating enzyme activity.

### Inhibition of SOD and CAT by high Cd^2+^

**Fig. 4:**
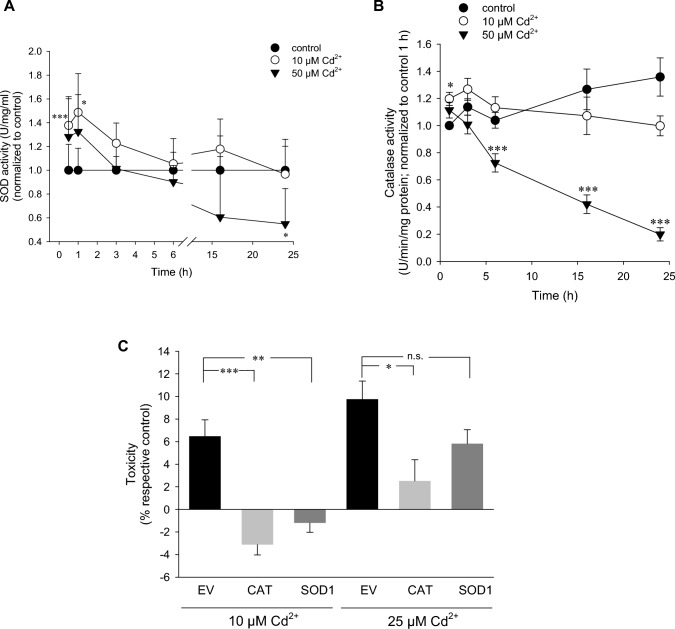
Inhibition of SOD and CAT by high Cd^2+^ contributes to H_2_O_2_ dominance. Time course of SOD (**A**) (*n* = 5–11) and CAT (**B**) (*n* = 18–19) activities after Cd^2+^ exposure. **C** Transient transfection of empty vector (EV), human *SOD1* or *CAT* plasmids for 24 h followed by 6 h Cd^2+^ exposure. Toxicity was determined by MTT assay (*n* = 4–5). Comparisons between Cd^2+^ treated and control samples were performed using one-way ANOVA and Holm–Sidak post hoc test at each timepoint

The antioxidative enzymes SOD and CAT are responsible for metabolizing superoxide anion to H_2_O_2_ and H_2_O_2_ to oxygen and water, respectively. Mammalians express three isoforms of SOD that are expressed in the cytosol (SOD1), mitochondria (SOD2) and extracellular space (SOD3). Conversely, only one *CAT* gene is present. As shown in Supplementary Figure 2A, *Sod1* (0.62 ± 0.23, *n* = 15) and *Sod2* (0.86 ± 0.20, *n* = 15) are highly expressed in PT cells. In contrast, very little *Sod3* mRNA is present (0.0001135 ± 0.0001138, *n* = 15). Though *Cat* mRNA is not as abundant as *Sod1* and *Sod2*, there is still a significant detectable level (0.17 ± 0.12, *n* = 15) (data not shown). To determine whether these enzymes are affected by Cd^2+^ and play a role in disrupting the balance in ROS/RNS, we first ascertained their enzymatic activities. SOD activity was significantly stimulated by 10 µM after 0.5–1 h (Fig. 4A), implying SOD enzymes are actively catalyzing dismutation of Cd^2+^-induced superoxide anions. SOD activity was also initially increased by 50 µM Cd^2+^, but did not reach statistical significance (Fig. 4A). Supporting data demonstrated *Sod1* mRNA expression was attenuated by 10 µM Cd^2+^ after 18 h (Supplementary Figure 2B), further pointing towards maintenance of superoxide anion levels due to diminished dismutation by SODs. High Cd^2+^ had the opposite effect: *Sod1* mRNA was significantly augmented after 3 h (Supplementary Figure 2B), though no increase in activity could be detected at this timepoint (Fig. 4A), and could not compensate for long-term downturn in SOD activity (by ~50% after 24 h) (Fig. 4A), possibly through interference of SOD1 protein stabilization by Cd^2+^ and/or a shift in zinc availability through upregulation of metallothionein (Polykretis et al. 2019). *Sod2* mRNA was unaffected by Cd^2+^ and *Sod3* mRNA was increased by high Cd^2+^ after 2 h (data not shown).

As one of the major H_2_O_2_ metabolizing enzymes, we investigated the role of catalase. Whilst all Cd^2+^ concentrations tested could increase catalase activity after 3 h (Supplementary Figure 2C), time course studies indicated elevated catalase activity after 1–3 h followed by inhibition of catalase at later timepoints, particularly at 50 µM Cd^2+^ (Fig. 4B). These observations are supported by qPCR (Supplementary Figure 2D) and immunoblotting (Supplementary Figure 2E, F). Conversely, 10 µM Cd^2+^ induced catalase protein expression is significant after 1 h, maintained until 6 h, and thereafter it decreases by ~20% compared to non-treated controls (Supplementary Figure 2E, F). Due to the strong inhibition of high Cd^2+^ on catalase expression and activity, it would be plausible to postulate H_2_O_2_ as the predominate ROS (Figs. 1F, 2B).

The impact of SOD and CAT expression and activity by Cd^2+^ on cytotoxicity was investigated. Heterologous expression of human *SOD1* or *CAT* was confirmed by PCR (Supplementary Figure 2G). CAT overexpression significantly abolished toxicity by both 10 µM and 25 µM Cd^2+^ after 6 h, as determined by MTT assay (Fig. 4C), confirming previous studies (Lee et al. 2012). In contrast, SOD1 also abolished cytotoxicity by 10 µM Cd^2+^ but only reduced 25 µM Cd^2+^ toxicity by about one third and did not reach statistical significance (Fig. 4C). Since superoxide anions are dismutated to H_2_O_2_, a catalase substrate, these observations align with the hypothesis that superoxide anion generation occurs only at lower Cd^2+^, whereas H_2_O_2_ is present at all Cd^2+^ concentrations tested. Taken together, distinct ROS/RNS species by Cd^2+^ participates in recruiting downstream signaling pathways and determining cell fate.

### NOX4 expression contributes to Cd^2+^-concentration-specific ROS

**Fig. 5:**
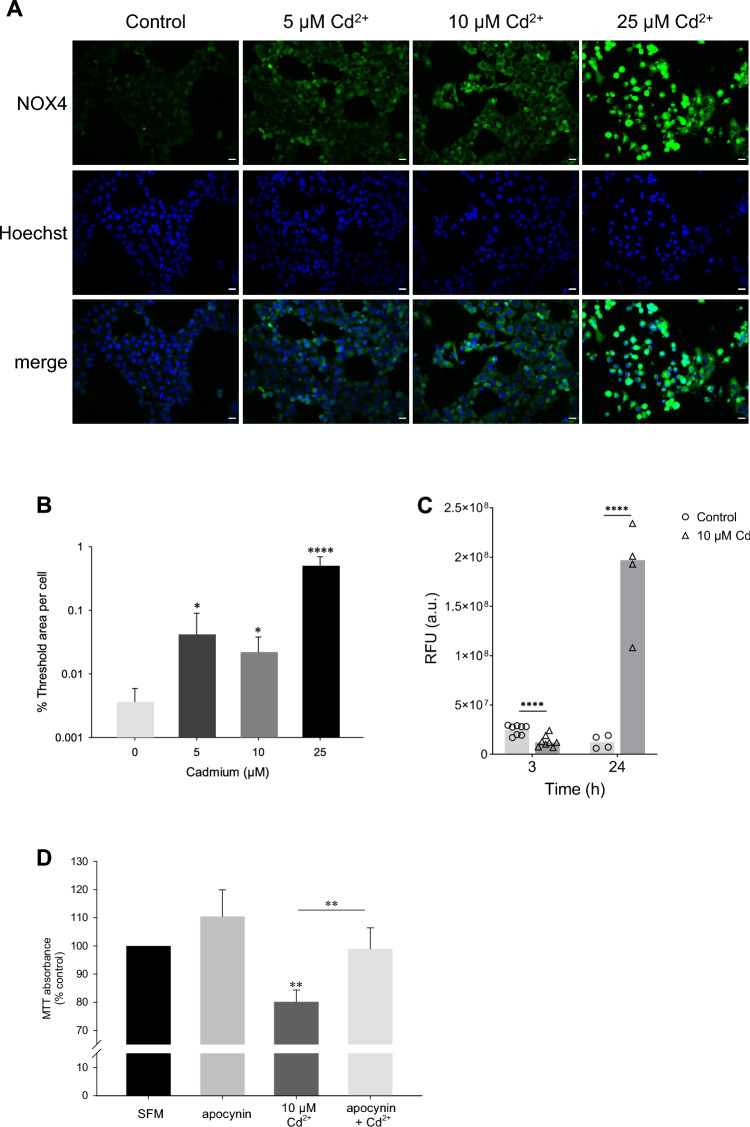
Kinetics of NOX4 expression by Cd^2+^ underscore generation of distinct ROS species. **A** Immunofluorescence staining for Nox4 in Cd^2+^-treated cells after 24 h. Scale bar = 20 µm. Images are representative of 4 independent experiments. **B** Quantitative analysis of thresholded images (from 3382 cells) from experiments in (**A**). **C** Fluorescence intensity analysis of NOX4 immunofluorescence after 10 µM Cd^2+^ exposure (*n* = 4–8). **D** MTT cell viability assay of WKPT-0293 Cl.2 cells exposed to 10 µM Cd^2+^ for 6 h pre-treated with 10 µM apocynin for 1 h (*n* = 4–5). Statistical analyses were performed using one-way ANOVA with Dunn’s test compares Cd^2+^ treated to control cells (**B**) or multiple comparisons with Holm–Sidak posthoc test (**D**). Unpaired two-tailed *t* test compares Cd^2+^-treated cells to controls (**C**)

The majority of intracellular ROS stems from enzymatic reactions as part of the ETC located in mitochondria. Assuming modest changes in antioxidative capacity, the simplest mechanism for Cd^2+^ induced ROS would be altered activity of the respiratory complexes, and therefore ROS production, in mitochondria, as reported earlier (Wang et al. 2004). Furthermore, we have previously shown that Cd^2+^ is taken up into mitochondria via the Ca^2+^ uniporter and induces mitochondrial swelling (Lee et al. 2005a). However, since Cd^2+^ has no impact on total cellular ATP levels, even at Cd^2+^ concentrations and timepoints, where ROS are elevated (Nair et al. 2015a), we hypothesized NOXs could be regulated by Cd^2+^ and contribute to ROS generation, as previously suggested (Thijssen et al. 2007). NOX isoform analysis at the mRNA level (Supplementary Figure 3A) confirmed previous observations evidencing *Nox4* as the predominant isoform in renal tubular cells with highest expression in the PT (Sedeek et al. 2013). In contrast to NOX1 and NOX2, which produce superoxide anions, NOX4 generates H_2_O_2_ and is also found in mitochondrial membranes (Altenhofer et al. 2012; Bedard and Krause 2007). At 10 µM Cd^2+^, *Nox4* mRNA is significantly decreased from 6 h onwards, whereas 50 µM Cd^2+^ induced *Nox4* mRNA at early exposure times (15–60 min) and negatively impacted *Nox4* mRNA from 3 h (Supplementary Figure 3B, C). At the protein level, NOX4 immunostaining revealed sustained augmented NOX4 signals at all Cd^2+^ concentrations tested after 24 h (Fig. 5A). Quantitative analysis evidenced 11.6, 6.1 and 138.2-fold increase in NOX4 at 5, 10 and 25 µM Cd^2+^, respectively (Fig. 5B). NOX4 was found intracellularly with punctate distribution suggesting localization to mitochondria as previously described (Altenhofer et al. 2012). To this end, co-staining for the mitochondrial protein cytochrome *c* revealed positive colocalization between NOX4 and cytochrome *c* following 10–25 µM Cd^2+^ after 24 h (data not shown). At 25 µM Cd^2+^, rounding of cells indicative of cell death were observed (Fig. 5A). Interestingly, time course studies with 10 µM Cd^2+^ evidenced decreased NOX4 protein at 3 h (by ~0.6-fold) yet a strong increase at 24 h (by ~15-fold) (Fig. 5C). Finally, the disputed NOX inhibitor and general ROS scavenger apocynin abolished the decrease in cell viability by 10 µM Cd^2+^ after 6 h (Fig. 5D), whereas the NOX inhibitor (Reis et al. 2020) diphenylene iodonium (DPI) did not (Supplementary Figure 3D). Taken together, the above data suggest early downregulation of NOX4 by 10 µM Cd^2+^, thus precluding an initial rise in H_2_O_2_ levels and supporting the shift to superoxide anion as the predominant ROS/RNS at lower Cd^2+^ concentrations. As time progresses, or at higher Cd^2+^, NOX4 suppression subsides permitting its contribution to toxic levels of H_2_O_2_.

## Discussion

In the present study, we have investigated the role of different chemical species of ROS in mediating varying levels of Cd^2+^ stress and toxicity in renal PT cells, the main site of Cd^2+^ accumulation in the kidney. The Cd^2+^ concentrations employed in this study serve to distinguish low–moderate and high Cd^2+^ as Cd^2+^ accumulation and Cd^2+^-associated disease progresses. Until now, the role of ROS has been established in Cd^2+^ signaling though in an undifferentiated view through the use of general antioxidants. Here, we report distinct ROS profiles by Cd^2+^, depending on its concentration, that likely influence the downstream pathways, which are recruited. Our results suggest mild-to-moderate toxicity and an adaptive response are attributed to O_2_^•−^, whereas supraphysiological levels of H_2_O_2_ surpass the threshold for adaptation and elicit a detrimental cellular response.

### ROS/RNS species in cell signalling

Both O_2_^•−^ and H_2_O_2_ are major physiological ROS species derived mainly from mitochondria and NOX enzymes (Sies et al. 2022; Sies and Jones 2020). In the ETC, O_2_^•−^ are generated by complexes I and III. Due to its charge, relatively short half-life and lower abundance, O_2_^•−^ is thought to transmit signals from local hotspots, such as within the foldings of mitochondrial cristae, or through oxidative bursts from single mitochondria (Booth et al. 2021). Interestingly, the abundant outer mitochondrial membrane (OMM) voltage-dependent anion channel VDAC (Han et al. 2003; Tikunov et al. 2010) and translocase of the outer membrane (TOM) complex (Budzinska et al. 2009) are postulated to control the release of O_2_^•−^ to the cytosol.

Aside from its direct effects, O_2_^•−^ is dismutated to H_2_O_2_ by matrix SODs or the peroxiredoxin system in the intermembrane space (IMS). Mitochondrial IMS H_2_O_2_ is also generated by NOX4. Cytosolic and peroxisomal catalases readily metabolize H_2_O_2_ to O_2_ and water.

### Cadmium impact on ROS generating and ROS metabolizing enzymes

**Fig. 6:**
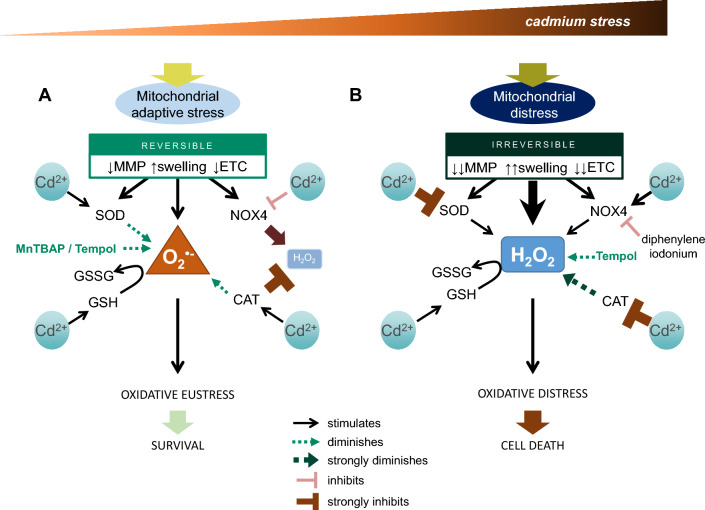
Model for distinct oxidative stress profiles by low and high cadmium. **A** Under low–moderate Cd^2+^ stress, adaptive responses are initiated. Reversible mitochondrial changes in mitochondrial membrane potential (MMP), swelling and electron transport chain (ETC) as well as direct Cd^2+^ effects on superoxide dismutases (SOD), catalase (CAT), NAPDH oxidase 4 (NOX4), reduced glutathione (GSH) and oxidized glutathione (GSSG) facilitate predominance of superoxide anions (O_2_^•−^) and preventing detrimental H_2_O_2_ levels. **B** As Cd^2+^ stress persists or increases, mitochondria become distressed leading to irreversible dysfunction. Moreover, high Cd^2+^ upregulates NOX4 and inhibits SOD and CAT culminating in augmented H_2_O_2_ levels, which engages an oxidative distress response that may lead to cell death. Inhibitors shown are MnTBAP (SOD mimetic), Tempol (SOD mimetic and free radical scavenger), and diphenylene iodonium (NOX4 inhibitor). For further details, see main text

It is well established that Cd^2+^ disrupts the balance in ROS/RNS-generating and ROS/RNS-reducing mechanisms [reviewed in (Thévenod et al. 2020)]: Activation of NOX4 or ETC complexes (particularly complexes I and III) augment ROS/RNS production, whereas Cd^2+^ binding to GSH and vitamins (Ribas et al. 2014; Vaskova et al. 2023) or inhibition of antioxidant enzymes, such as SODs and CAT, (Cuypers et al. 2010; Jena et al. 2023) reduces the cellular antioxidative capacity, culminating in oxidative distress with higher Cd^2+^ concentrations or with prolonged periods of exposure. In addition, expression levels of endogenous antioxidants are usually enhanced by Cd^2+^. The current study highlights differences in Cd^2+^ impact on the oxidative and antioxidative machinery depending on the Cd^2+^ concentration applied and exposure time. Low to moderate Cd^2+^ concentrations, which aim to recapitulate a real-world situation of slow accumulation of Cd^2+^, favours O_2_^•−^ and impedes initial H_2_O_2_ generation (Figs. 3, 4, 6A). In contrast, higher Cd^2+^ concentrations, such as during acute toxicity, utilize non-O_2_^•−^ ROS, namely H_2_O_2_, to engage signaling pathways (Figs. 1, 2, 6B). In fact, the generated H_2_O_2_ levels are sufficient to elicit oxidative distress by further inhibiting antioxidative enzyme activity as well as blocking their expression, and culminating in cell death (Fig. 4). Though SODs and CAT in the context of Cd^2+^ toxicity are commonly investigated, less is known about other key enzymes in ROS/RNS metabolism. Changes in GPx would greatly impact GSH antioxidative capacity because GPx catalyzes the oxidation of GSH to GSSG by H_2_O_2_ (Vaskova et al. 2023). In preliminary experiments, 10–50 µM Cd^2+^ after 3 h did not affect mRNA levels of *Gpx1*, *Gpx2* and *Gpx4* (data not shown). On the other hand, the thioredoxin–peroxiredoxin system directly governs H_2_O_2_ availability (Go and Jones 2013) and could underline distinct ROS profiles by Cd^2+^. Activation of thioredoxins by Cd^2+^ (Go et al. 2013; Hansen et al. 2006; Sakurai et al. 2005) suggests H_2_O_2_ is diminished and supports our claim that O_2_^•−^ is preferred at low and early Cd^2+^.

A pivotal factor is catalase expression, subcellular distribution and activity. Intriguingly, 10 µM Cd^2+^ transiently increased catalase activity yet it was strongly inhibited by 50 µM Cd^2+^ (Fig. 4A). How Cd^2+^ may affect catalase activity is unclear. It could be speculated that Cd^2+^ displaces or interacts with iron in the heme group or reacts with key thiol groups or amino acids in the active site, e.g., histidine (Permyakov 2021).

### Impact of different ROS/RNS species on cadmium cell death and survival signaling

The extent of oxidative stress dictates downstream cellular responses. Mild oxidant elevation, or oxidative eustress, can be viewed as a physiological signalling system that relays information about the cellular or organellar oxidative status, such that timely adaptive changes can be engaged (Lennicke and Cocheme 2021; Rhee 1999; Sies and Jones 2020). In contrast, supra-physiological oxidant overload (>100 nM H_2_O_2_) could lead to progressively stronger activation of the adaptive response. When the oxidant overload cannot be rescued, activation of cell death pathways causes the cell to succumb (Brand 2020; Sies and Jones 2020).

How might different Cd^2+^ concentrations, and hence different ROS/RNS, affect downstream signalling pathways governing cellular life and death decisions (Fig. 6)? Intracellular mitochondrial O_2_^•−^ signals affect Fe–S clusters, such as those within the ETC (Sies and Jones 2020). Therefore, it would be plausible to postulate low–moderate Cd^2+^ concentrations act on mitochondria to induce small oxidant increases by affecting ETC membrane stability, which permits the cell to initiate an adaptive response, such as transient mitochondrial swelling, activation of protective transcription factors (e.g. nuclear factor-kappa B [NF-kB], nuclear factor erythroid 2-related factor 2 [Nrf2]) and/or metabolic changes (autophagy induction) (Fig. 6A). Cd^2+^-induced O_2_^•−^, but not H_2_O_2_, also initiates ER stress leading to engagement of the adaptive unfolded protein response (Yokouchi et al. 2008). As Cd^2+^ stress intensifies, the dominant ROS is shifted to H_2_O_2_ and the adaptive response prevails, but additional responses that progress towards cellular toxicity are also initiated, for example, reduction in antioxidative capacity, activation of H_2_O_2_-generating NOX4 (Thijssen et al. 2007 and this study), ER stress and the unfolded protein response (Yokouchi et al. 2008), lysosomal instability (Lee et al. 2017), irreversible mitochondrial swelling (Lee et al. 2005b; Fig. 6B).

In summary, Cd^2+^ initiates distinct ROS/RNS profiles in a concentration-dependent manner, directing the cellular decisions between toxicity and survival, and ultimately impacting cellular consequence.

### Supplementary Information

Below is the link to the electronic supplementary material.

Supplementary file1 (DOCX 278 KB)
