# Supplementary material for: Distinct concentration-dependent oxidative stress profiles by cadmium in a rat kidney proximal tubule cell line
Source: Arch Toxicol. 2024 Jan 30;98(4):1043–59. doi: 10.1007/s00204-023-03677-z (PMC10944451; doi:10.1007/s00204-023-03677-z)
Supplement: Supplementary file 1 — Supplementary file1 (DOCX 278 KB) [file 204_2023_3677_MOESM1_ESM.docx]

**Supplementary Figure 1**

DHE oxidation products after 1 h 10 µM Cd^2+^. Cells were lysed after Cd^2+^ and DHE loading. Oxidation products ethidium (less superoxide specific) and 2-hydroxyethidium (superoxide-specific) were determined at 380/590 nm and 535/590 nm, respectively (n = 8-12).

**Supplementary Figure 2**

(A) Relative mRNA expression of endogenous superoxide dismutase isoforms (*Sod1-3*) in non-treated WKPT-0293 Cl.2 cells determined by qPCR (n = 7-8). (B) Time course of *Sod1* mRNA. Cells were collected at each time point and subjected to qPCR analysis (n = 7) and compared after 18 h 10 µM Cd^2+^ or 3 h 50 µM Cd^2+^ exposure (n = 7). Unpaired two tailed t-test compares Cd^2+^-treated cells to controls. (C) Cd^2+^ concentration dependent catalase activity after 3 h in WKPT-0293 Cl.2 cells (n = 4). (D) Time course of *Cat* mRNA expression after Cd^2+^ treatment determined by qPCR (n = 7). (E) Representative catalase immunoblot and Coomassie staining of Cd^2+^ treated cells. (F) Densitometry analysis of catalase signals after correction for protein loading (n = 8). Comparisons between Cd^2+^ treated and control samples were performed using one-way ANOVA and Holm-Sidak posthoc test. (G) Expression of human *SOD1* and *CAT* in transiently transfected WKPT-0293 Cl.2 cells after 24 h using semi-quantitative reverse transcriptase PCR. *Gapdh* serves as loading control. Image is representative of 3 independent experiments.

**Supplementary Figure 3**

(A) Relative mRNA expression of NADPH oxidase isoforms in non-treated WKPT-0293 Cl.2 cells determined by qPCR (n = 8). (B) Time course of *Nox4* mRNA. Cells were collected at each time point and subjected to qPCR analysis (n = 5-6). Comparison of *Nox4* mRNA expression after 18 h 10 µM Cd^2+^ or 0.5 h 50 µM Cd^2+^ exposure (n = 5-6). * p< 0.05, ** p< 0.01 by unpaired two-tailed Student’s t-test. (C)Time course of *Nox4* mRNA expression after Cd^2+^ treatment determined by qPCR (n = 9). (D) MTT cell viability assay of WKPT-0293 Cl.2 cells exposed to 10 µM Cd^2+^ for 6 h pre-treated with 1 nM diphenylene iodonium (DPI) for 1 h (n = 4-5). Comparisons between Cd^2+^ treated and control samples were performed using one-way ANOVA and Holm-Sidak post hoc test.

**Supplementary Table 1 Primers for quantitative real-time PCR**

| Gene | NCBI Accession Number | Forward primer (5' → 3') | Reverse primer (5' → 3') | Amplicon size (bp) | Reference |
| --- | --- | --- | --- | --- | --- |
| *Cat* | NM_012520 | GCGAATGGAGAGGCAGTGTAC | GAGTGACGTTGTCTTCATTAGCACTG | 121 | NCBI PrimerBLAST |
| *Sod1* | NM_017050 | GGATGAAGAGAGGCATGTT | TCATCTTGTTTCTCGTGGAC | 91 | NCBI PrimerBLAST |
| *Nox4* | NM_053524.1 | ACCAGATGTTGGGCCTAGGATTGT | AGTTCACTGAGAAGTTCAGGGCGT | 261 | NCBI PrimerBLAST |
| *Gapdh* | NM_017008.4 | AGGGCTCATGACCACAGT | TGCAGGGATGATGTTCTG | 112 | NCBI PrimerBLAST |
| *Ywhaz* | NM_013011.3 | CAAGCATACCAAGAAGCATTTGA | GGGCCAGACCCAGTCTGA | 76 | (Nair et al. 2015b) |
| *Actb* | NM_031144.2 | AGATCAAGATCATTGCTCCTC | ACTCATCGTACTCCTGCTTG | 117 | NCBI PrimerBLAST |

**Supplementary Table 2 Primers for reverse transcriptase semi-quantitative PCR**

| Gene | NCBI Accession Number | Forward primer (5' → 3') | Reverse primer (5' → 3') | Amplicon size (bp) | Reference |
| --- | --- | --- | --- | --- | --- |
| Human *CAT* | NM_001752 | CATTCGATCTCACCAAGGTTTGGCC | CCTGTGAACTGTCCCTACCGTGCT | 257 | (Kenney et al. 2005) |
| Human *SOD1* | AY049787 | AGTGCAGGGCATCATCAATTTCGAGCAG | GATCTCACTCTCAGGAGACCATTGCATC | 298 | (Kenney et al. 2005) |
| Rat *Gapdh* | NM_017008.4 | AATGCATCCTGCACCACCAACTGC | GCGGCATGTCAGATCCACAACGG | 300 | Primer 3 |
